# Mammary microbiome of lactating organic dairy cows varies by time, tissue site, and infection status

**DOI:** 10.1371/journal.pone.0225001

**Published:** 2019-11-14

**Authors:** Tucker Andrews, Deborah A. Neher, Thomas R. Weicht, John W. Barlow

**Affiliations:** 1 Department of Plant and Soil Science, University of Vermont, Burlington, Vermont, United States of America; 2 Department of Animal and Veterinary Sciences, University of Vermont, Burlington, Vermont, United States of America; University of Illinois, UNITED STATES

## Abstract

Infections of the cow udder leading to mastitis and reducing milk quality are a critical challenge facing all dairy farmers. Mastitis may be linked to the ecological disruption of an endogenous mammary microbial community, suggesting an ecosystems approach to management and prevention of this disease. The teat end skin represents a first point of host contact with mastitis pathogens and may offer an opportunity for microbially mediated resistance to infection, yet we know little about the microbial community of teat end skin or its potential interaction with the microbial community of intramammary milk of organic dairy cattle. High-throughput sequencing of marker genes for bacterial and fungal communities was used to characterize the skin and milk microbiome of cows with both a healthy and infected gland (i.e., udder quarter) and to assess the sharing of microbial DNA between these tissue habitat sites. The mammary microbiome varied among cows, through time, and between skin and milk. Microbiomes of milk from healthy and infected quarters reflected a diverse group of microbial DNA sequences, though milk had far fewer operational taxonomic units (OTUs) than skin. Milk microbiomes of infected quarters were generally more variable than healthy quarters and were frequently dominated by a single OTU; teat end skin microbiomes were relatively similar between healthy and infected quarters. Commonly occurring genera that were shared between skin and milk of infected glands included *Staphylococcus spp*. bacteria and *Debaryomyces spp*. fungi. Commonly occurring genera that were shared between skin and milk of healthy glands included bacteria SMB53 (Clostridiaceae) and *Penicillium spp*. fungi. Results support an ecological interpretation of the mammary gland and the notion that mastitis can be described as a dysbiosis, an imbalance of the healthy mammary gland microbiome.

## Introduction

Mastitis remains one of the most common and costly health concerns of dairy cattle in the United States (US) and globally. In the northeastern US, organic dairy farmers identify mastitis as a top animal health challenge area and mastitis control as a key research priority [1]. Prevention is critical to limiting mastitis, particularly on organic dairy farms, where efficacy of products approved to treat infections in organic cattle is limited [2]. Animal housing, bedding material, dairy facilities, and milking hygiene practices influence the cow’s exposure to environmental bacteria and fungi and may influence risk of mastitis caused by opportunistic pathogens [3–5]. The microbiota of both teat skin and milk may reflect environmental factors [3, 5], yet the link between teat skin microbial community structure and risk of intramammary infection (IMI) is currently unknown. In theory, the ability to foster a commensal mammary microbiome that limits IMI risk would benefit both organic and conventional dairy farms. Therefore, understanding the existence and potential importance of a teat apex skin microbiota deserves attention [6].

Genetic sequencing of the host microbiome is redefining the traditional knowledge of the mammalian relationship with microbes, expanding our understanding of diversity and abundance of microbes observed in healthy mammalian tissue sites, and evincing a paradigm shift away from describing infection as a two-way host-pathogen interaction toward a community ecology perspective [7, 8]. This hypothesis posits that an infection event is linked to an ecological disruption of the endogenous microbial community of a healthy host habitat. The resulting observed imbalance of the microbiome relative to the healthy state has been described as “dysbiosis” [6, 7, 9].

Culture-independent assays of the mammary microbiome suggest a diversity and presence of microbes in milk from healthy and infected glands far greater than previously described [10–15], leading some researchers to suggest that mastitis can be understood as a dysbiosis [10, 11]. This is not without controversy, especially the presence of a healthy milk or intramammary microbiome [6]. For decades, mastitis researchers have described the healthy intramammary environment as sterile, with the introduction of microbes leading to some degree of local or systemic inflammation [6]. Even accepting the sterile intramammary lumen argument, an ecological interpretation of the mammary microbiota is necessitated by the diversity of microbes over multiple host habitats (teat skin, teat apex skin, teat canal epithelium, teat cistern) with potential transmission of pathogens among habitats [16].

The possibility of commensal or beneficial teat microbiota influencing mastitis risk is not without precedent [17]. Skin is an ecosystem with diverse and distinct habitats supporting a wide array of microbiota that provide critical functions to the host [18]. Likewise, recent culture-independent assays of the bovine teat apex and teat canal suggest these sites are habitats for diverse microbiota [12, 19]. An ecological approach to mastitis epidemiology, therefore, integrates microbiota data across multiple habitats to understand how potential pathogens fit into the greater microbial ecosystem [20].

In this study, high-throughput sequencing of marker genes was used to compare composition of bacterial and fungal communities of teat end skin and milk from mammary glands of organic dairy cattle with and without subclinical mastitis due to intramammary bacterial infections. Putative communities were characterized and relationships explored between two host-habitats (teat end surface and milk collected directly from the teat cistern) of dairy cattle mammary glands in the infected and healthy states.

## Methods

Here, we adopt the term “habitat state” to generally describe the two habitats (teat apex skin and teat cistern milk) in either the healthy or infected state. Thus, the four habitat states sampled were teat end skin and teat cistern milk of a mammary gland (individual udder quarter) observed in either a healthy or infected state. For brevity, we will refer to samples from these habitat states as “infected milk”, “healthy milk”, “infected teat”, and “healthy teat”. A more precise but less concise description of the samples would be “cisternal milk sample from a gland defined as infected” for infected milk, or “teat end skin swab sample from a gland defined as uninfected” for healthy teat, etc. The *a priori* design of the observational study was to collect teat swab and milk samples from healthy and infected mammary quarters during the grazing season on a single dairy herd over two years to compare composition of bacterial and fungal communities in these four sample types.

### Experimental design and cow selection

A commercial, certified organic dairy herd was sampled in June, July, and August of 2015 and 2016. In year one, sampling occurred twice per month, the first sampling served to identify animals and glands to be sampled more extensively later in the month. For the first monthly sample, all lactating glands of all cows were sampled in duplicate using established aseptic technique, followed by bacteriologic culture using established procedures [21]. Briefly, 10 μl of each milk sample was spread on a tryptic soy agar with 5% sheep blood plate and incubated aerobically for 48 hours. An intramammary infection (IMI) was defined for a gland if the same bacterial organism was isolated from the duplicate milk cultures obtained on the same day. Glands with three or more distinct colony morphologies were considered contaminated and removed from analysis. Milk somatic cell count (SCC; cells/ml milk) was measured using a Somacount 150 instrument (Bentley Instruments, Chaska, MN, USA). Mastitis was defined as a gland with SCC ≥ 200,000 cells/ml and was further defined as clinical if there were associated clinical signs, or subclinical (asymptomatic) if there was no evidence of clinical signs of mastitis for the gland or cow. Glands that had a SCC less than 100,000 cells/ml and no IMI were designated as healthy. Cows were selected for resampling on the second visit if subclinical mastitis and IMI was detected in at least one quarter, and one or more other quarters of the same cow were defined as healthy. Cows with evidence of clinical mastitis events within the 14 days prior to sampling were excluded from the study in that sampling month.

On the second monthly visit, approximately two weeks later, a series of milk and teat end skin swabs were obtained from at least one infected gland with subclinical mastitis, and one healthy gland from selected cows. Disposable nitrile exam gloves were worn during sample collection. Debris and gross visual skin contamination were removed from the udder, teat barrel and teat end by wiping with a dry disposable paper-towel. Prior to application of any pre-milking teat disinfectant, teat ends were swabbed using a nylon flocked swab (FloQSwab 502CS01, Copan Diagnostics Inc., Murrieta, CA, USA) moistened in sterile molecular grade DNA-free water. The distal portion of the swabs were returned to 5 ml molecular grade water in a 15 ml conical tube by breaking the shaft at the break-point, and swab samples were immediately placed on ice for transport to the laboratory. The teat end was then cleaned and disinfected using a series of 10x10 cm cotton gauze pads moistened in 70% ethanol. An initial fore-milk sample was hand stripped from the gland and discarded. Subsequently, milk samples were collected in duplicate by hand stripping directly into a 10 ml snap cap tube (conventionally collected milk sample). Teat cistern milk samples were then collected using a modification of the teat cannulation technique reported previously [22]. In this modification, after the sterile plastic 34 mm teat cannula (J-12 Teat infusion Cannula, Jorgensen Laboratories, Loveland, CO, USA) was inserted into the teat canal, a sterile closed tip semi-rigid polypropylene 14 cm catheter (Argyle Tom Cat Catheter, Medtronic Animal Health, Minneapolis, MN, USA) was passed through the teat cannula approximately 10 cm into the teat cistern, and 5 ml of cisternal milk was aspirated directly from the teat cistern using a syringe attached to the catheter.

Control “sham” teat swab samples were collected in the barn each month in 2016, by removing swabs from individual wrapping, moistened in sterile molecular grade water, holding in the air adjacent to a cow flank for approximately 10 seconds, and returned to the transport vial. Aliquots of the sterile molecular grade water, not exposed to the barn environment, were also processed as parallel negative control samples in the laboratory once in 2015 and twice in 2016.

Conventionally collected milk samples were cultured and SCC measured as described previously for the first monthly sample to confirm infection and mastitis status. For initial processing, teat end swab samples were homogenized in a multi-tube platform vortex mixer at 2,500 rpm for 5 minutes, the swab was removed, and 1 ml aliquots of suspensions stored at -20°C until further processing for culture independent analysis. Cisternal milk samples were divided into 1 ml aliquots and stored at -20°C until DNA was extracted for marker gene sequencing.

Cows were selected for the second monthly sampling to represent the most common genera of pathogens causing IMI in the herd. From the selected cows for the second monthly sampling (6 to 12 per month), at least six cows were selected randomly for targeted DNA sequencing of microbial community from teat end swab and teat cistern milk samples. Sequencing was performed on one healthy and one infected gland per cow for each cow enrolled each month. If more than one healthy or mastitic gland was identified within a cow, preference was given to glands that would create an even distribution of udder gland position (i.e., left-front, right-hind) across all cows in each month. In year two (2016), no screening was conducted prior to gland sampling in each month; instead, cows identified in 2015 were tracked for a second year. As in 2015, gland SCC was measured, culture was performed in duplicate to identify pathogens; milk and teat swab samples from one uninfected healthy and one infected mastitic gland from a random subset of sampled cows were submitted for sequencing of bacterial and fungal marker genes. If a cow that had been sampled previously was removed from the herd or became unavailable for sampling, another cow was chosen randomly from the screened group as a replacement. Individual glands that changed status from uninfected to subclinical mastitis with IMI (*n* = 10) were removed from the data. A total of 114 samples from 13 cows were retained for analysis, including 28 infected milk, 28 healthy milk, 29 infected teat and 29 healthy teat samples. This study was conducted in accordance with the recommendations in the Guide for the Care and Use of Agricultural Animals in Research and Teaching of the Federation of Animal Science Societies. The protocol was approved by the Institutional Animal Care and Use Committee of the University of Vermont (Protocol Number: 15–039).

### Farm description and animal husbandry

The study was conducted at a 150-cow certified organic pasture-based dairy that feeds an all grass and hay ration. The herd is a mix of Holstein and Jersey breeds that yield a farmer reported mean of 20 kg milk per cow per day. During the study periods, cows spent the majority of their time on pasture, returning to the barn to be milked twice per day with a high-line system in tie-stalls, with concrete cubical flooring bedded with sawdust or wood shavings.

In the first year of sampling (2015), the farm applied no pre- or post-milking teat end disinfection, and for at least one year prior to the initiation of the study pre-milking hygiene consisted of dry wiping cows with disposable paper towels to remove debris and gross contamination. In September 2015, the farm reinstituting the use of pre- and post-milking teat disinfection. During the second sampling year (2016) pre-milking hygiene consisted of dry wiping cow teats with disposable paper towels to remove debris and gross contamination, fore-stripping glands, application of a pre-milking teat end disinfection, allowing for 60–90 seconds contact time, followed by removing teat disinfectant from all four teats with a single use disposable paper towel. The teat disinfectant used for both pre- and post-milking application was Quadra-Plex iodine liquid (IBA Inc., Millbury, MA, USA) containing 5% Nonylphenoxypolyethoxyethanol Iodine Complex (1.0% minimum titratable iodine), 10% emollients, and 85% other inert ingredients including buffering agents and surfactants (pH 5.5 at time of manufacture). All milking personnel routinely wore disposable nitrile gloves during all milking procedures in both years.

### Extraction, sequencing, taxonomic mapping, and data management

DNA was extracted from 1 ml aliquots of thawed milk and teat swab samples using the Qiagen PowerSoil Soil DNA Isolation kit (Germantown, MD) following the manufacturer’s instructions, and the methods described by Lauber *et al*. [23]. Specifically, bead tubes were heated to 65°C for 10 min, and then shaken horizontally for 2 min at maximum speed with the MoBio vortex adapter. The remaining steps were performed as directed by the manufacturer [23]. Extracted DNA samples were frozen at -80°C until shipment to the University of Colorado Next Generation Sequencing Facility (Boulder, CO) for PCR amplification, sequencing, initial data filtering and taxonomic reference mapping [24]. Samples from each year were sequenced in independent batches and raw sequences from both batches were pooled for binning and OTU assignment. Aliquots of sterile molecular grade water were also processed in parallel as DNA extraction negative control “blank” samples (one blank extraction control included with every set of 12 to 20 samples processed).

Extracted DNA was PCR-amplified using 515F/806R primers targeted for the V4 region of the 16S rRNA gene for bacteria and archaea, and fungal ITS1F and ITS2 primers to amplify the first rRNA internal transcribed spacer region (ITS1). Reactions were held at 94°C for 3 min to denature the DNA, with amplification proceeding for 35 cycles at 94°C for 45 s, 50°C for 60 s, 72°C for 90 s, and 10 min at 72°C, followed by a final extension of 10 min at 72°C. Negative (sham and blank) controls were included to test for contamination. Triplicate PCR reactions were pooled for each sample and amplicon concentrations were measured with a PicoGreen dsDNA assay (Life Technologies, Grand Island, NY). Sequencing was performed on an Illumina MiSeq (2 x 150 bp chemistry). Reads were merged, demultiplexed and quality-filtered (max expected error < 0.5) and chimera and singletons removed using UPARSE following the pipeline described previously [25]. Briefly, sequences were de-replicated, and a database of single representative sequences for each operational taxonomic unit (OTU) in the data were generated via UCLUST (version 7) [26] clustering at 97% nucleotide identity, and then reads from the entire data set were mapped back to the representative bacterial or fungal database to generate one OTU table for bacteria and one for fungi. Taxonomy was assigned to each OTU via the Greengenes v13.8 database for bacteria [27] and by sequence comparison to the UNITE v7.2 fungal ITS database for fungi [28]. All mitochondria and chloroplast OTUs were removed from the 16S rRNA gene OTU table prior to downstream analyses. Reference sequences, OTU counts, taxonomy, and associated metadata are publicly available at https://figshare.com/articles/Bovine_mammary_microbiome/7365008.

Library size (read counts per sample) was determined and rarefaction curves generated to assess depth of sequencing relative to unique OTUs on a sample basis using the rarecurve function in vegan [29]. Samples with less than 100 sequences were removed prior to using a proportional scaling approach to compare between samples with disparate library sizes [30]. OTU counts within each sample were expressed as a proportion of total sample counts and multiplied by the mean of all sample counts. This scaled value was rounded to obtain a whole number, eliminating OTUs with a scaled proportion less than 1.0. ITS and 16S rRNA sequence counts were normalized separately. Normalized OTU abundances were then converted to a relative abundance (RA) of the total number of sequences per sample.

### Statistical analysis

All analyses were performed using R software. Bray-Curtis dissimilarity matrices were generated to compare samples and non-metric multidimensional scaling was used to visually represent similarities between sampling times, habitats, and habitat states. Abundances were transformed as the square-root (*x*) before calculating dissimilarity. Permutational multivariate analysis of variance (PERMANOVA) was conducted using the adonis function in vegan [29] for multivariate analysis of main effects. For bacteria, models testing herd-level variation in the microbiome due to sampling year, sample type (teat or milk), and infection status were permuted within cow and restricted to a one-way time series to control for the effect of the host on the microbiome and relatedness due to repeated measures. Variation associated with the individual cow and sampling date within year were tested separately. Due to the relative paucity of fungal sequences resulting in fewer samples, permutations would have been overly restricted if the nested series design was maintained, thus free permutations were used to assess significance, and the paired design was not maintained. The homoscedasticity within groups was measured by the distance between sample locations in Euclidean space and their geometric centroid using the betadisper function in vegan [31]. For bacteria, a co-inertia analysis was performed using the cia function in the made4 package to assess global relative similarity (RV), a multivariate extension of the Pearson correlation coefficient, between healthy and infected milk and teat community matrices [32]. Differences in taxa abundance between sample types were analyzed by Kruskal-Wallis tests using the package mctoolsr (https://github.com/leffj/mctoolsr/). Pielou’s index (J), used to assess evenness of OTU abundances within a sample, was calculated as J = Shannon-Weaver index/log(species richness). *P* values generated from multiple tests were false discovery rate adjusted using p.adjust and are referred to as *fdr* in the text. Bartlett’s test was used to compare variance of diversity within sample type.

A point bi-serial correlation coefficient was calculated to identify OTUs associated positively with one or more habitat states using the indicspecies package [33]. OTUs with significant associations (*p* ≤ 0.05) were represented in a bipartite network generated in the Fruchterman-Reingold layout using the igraph package [34]. We used the concept of a “core microbiome” to assess which microbes persist in multiple habitat states [35]. The core microbiome was calculated for bacterial sequences in year 2015 using the mctoolsr package. OTUs were first subset by presence at any abundance within each habitat state; of this group, OTUs were retained that were observed in all animals, in at least 50% of observations from that animal; this group was further refined to include only OTUs that were observed in 25% of samples on each sampling date. This threshold retained OTUs that were observed in at least two of four habitat states. Hierarchical clustering of untransformed Bray-Curtis dissimilarity distances between taxa in each habitat state was performed using Ward’s minimum variance method via the hclust function [36] and taxa were sorted via the resulting dendrogram.

## Results

### Summary of sequencing

Library size range for 16S rRNA amplicons was 186–38791 sequences (read counts per sample, median 7103, mean 12844, std. dev. 14179); ITS range was 2–71762 sequences (read counts per sample, median 18739, mean 21012, std. dev. 16705). Library size for negative control sham swab and blank samples ranged from 23–5812 16S rRNA sequence reads and 1–811 ITS sequence reads; no consistent trends in OTU diversity were observed among control samples (S1 and S2 Figs). While 250 of the 281 OTUs observed in control samples were also observed in the skin swab and milk samples, the mean read number for OTUs in controls was 2% of the mean read number for OTUs in the skin swab and milk samples, suggesting limited contamination during collection and processing of swab and milk samples. Rarefaction curves illustrated that species-richness as a function of sample size approached an asymptote for the majority of samples (S3 Fig). After removing samples with less than 100 sequences and eliminating OTUs with a scaled proportion less than 1.0, 5328 of 6021 16S rRNA OTUs were retained, which represented a 16% loss of OTU richness in 2015, zero loss in 2016 and preserved all samples (*n* = 114). Normalization of ITS data retained 789 of 949 OTUs. Rarefaction analysis indicated that 2015 ITS samples did not have good coverage and 10 samples below the 100 sequence threshold were removed, representing a 15% loss of richness; a single sample from 2016 was removed representing a 20% loss of richness; *n* = 70 were retained.

### Bacterial communities (16S rRNA)

**Fig 1:**
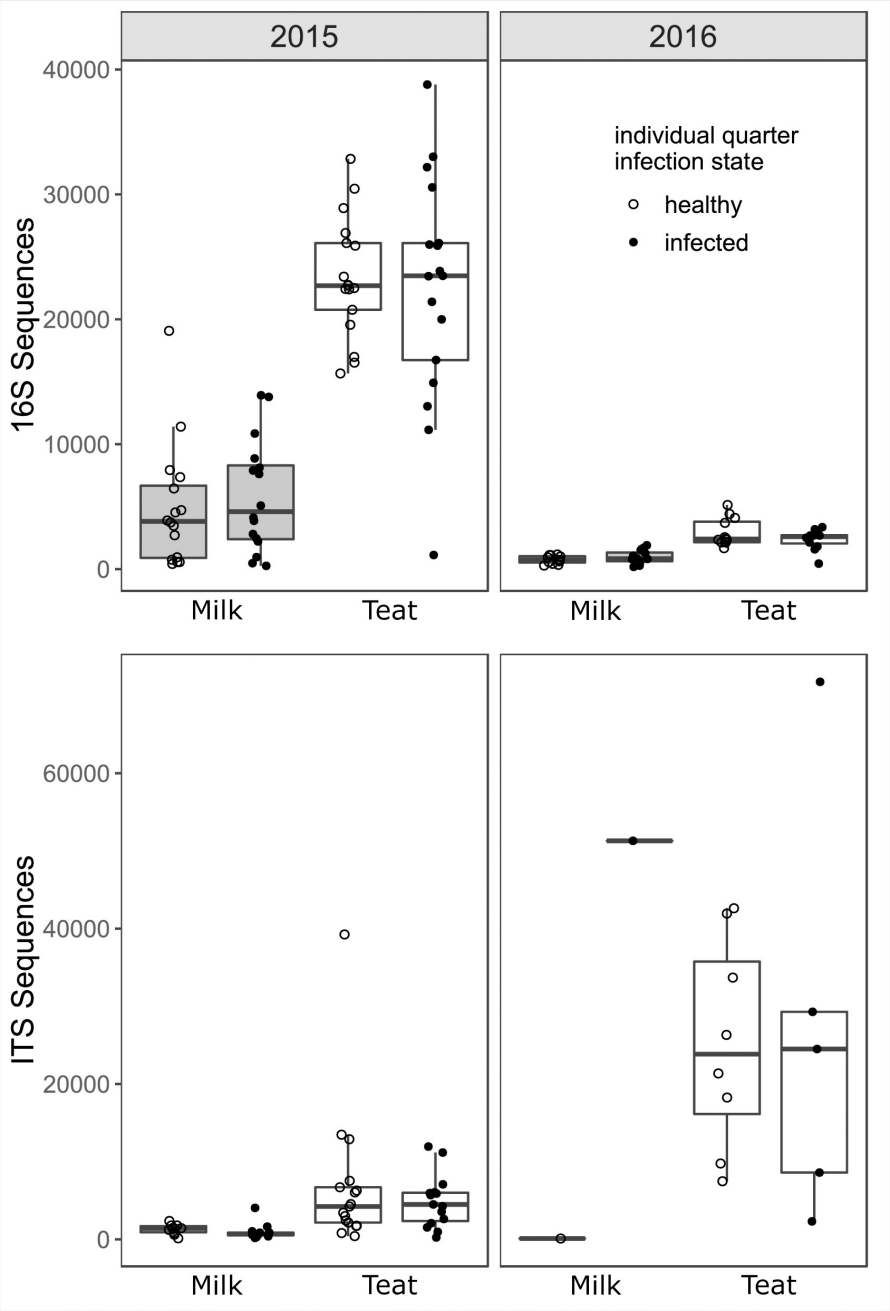
16S and ITS rRNA gene amplicon sequence counts by year and habitat state. Points represent gland samples prior to normalization. Upper plot: 16S rRNA sequences, *n* = 114. Sequence counts varied between years, median counts 2015 = 14484, median counts 2016 = 1752, *p* < 0.001). Lower plot: ITS sequence counts, *n* = 70. Note the difference in scale between the two plots.

**Fig 2:**
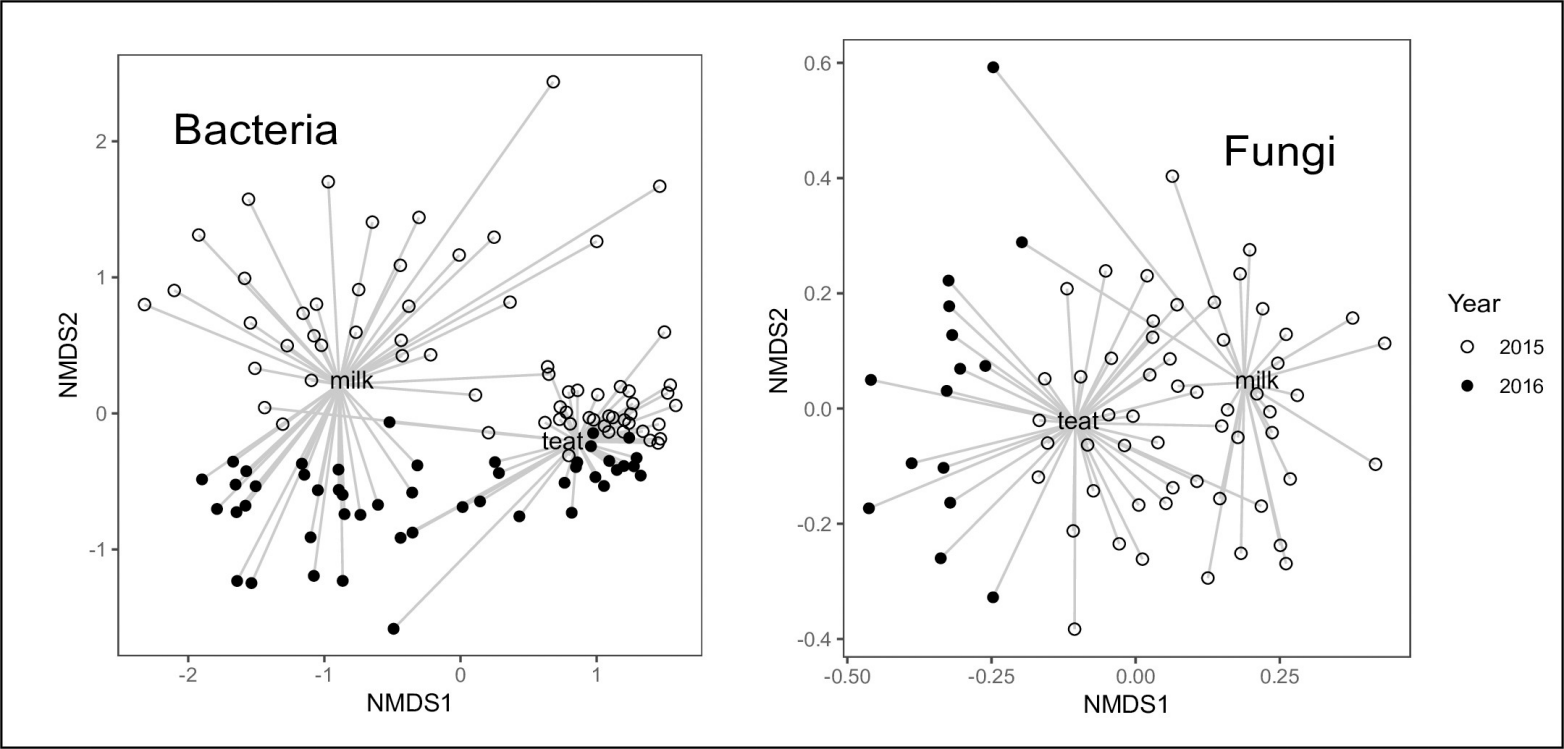
Bi-plot of Bray-Curtis dissimilarity of bacterial and fungal communities of intramammary milk and teat skin pooled across years. Points represent gland samples (n = 114 bacteria, n = 70 fungi) colored by sampling year. Lines represent distance to centroid of sample type. Abundances were transformed as square root prior to analysis.

**Fig 3:**
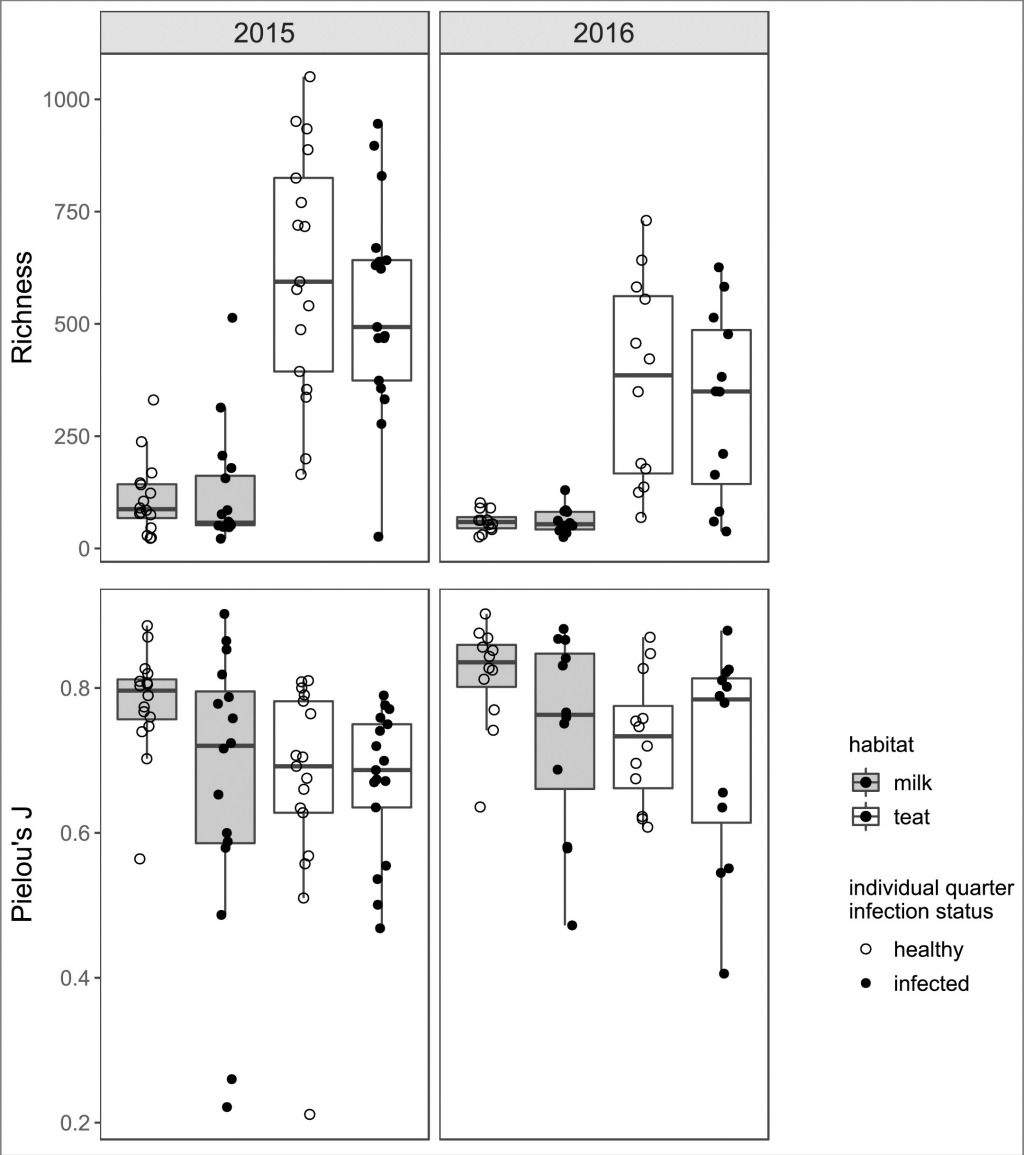
Bacterial OTU diversity as represented by richness and evenness of OTUs in each habitat state and year. Richness was calculated as number of OTUs; evenness was measured using Pielou's J (J = Shannon/log(richness)). Index values closer to 1.0 indicate increasingly even distributions of OTU abundances.

Variation in the mammary bacterial microbiome was associated with year and host habitat (milk or teat end) (Figs 1 and 2). Total sequences were fewer and less variable in milk than teat samples in both years, and fewer in 2016 than 2015 (Fig 1). More bacterial OTUs were detected in 2015 than 2016 (*p* = 0.002), and milk contained fewer OTUs than teat swabs in both years (*p* < 0.001) (Fig 3). Despite reduced richness, OTU abundances in 2016 samples were more evenly distributed than in 2015 (*p* = 0.027). Infected milk samples were more likely to have an uneven distribution of taxa abundances compared to healthy milk (*p* = 0.008), but minimal difference in richness (*p* = 0.556). Evenness and richness remained similar for both infected and healthy teat ends (*p* = 0.879, *p* = 0.331), although richness tended to decrease slightly on infected teat ends. OTUs observed in all habitat states comprised 8.1% of total OTUs (431 of 5328). Approximately 25% of OTUs in each habitat state were unique to that habitat state. Healthy milk had greater evenness of OTU abundances and less variation among glands than infected milk.

**Fig 4:**
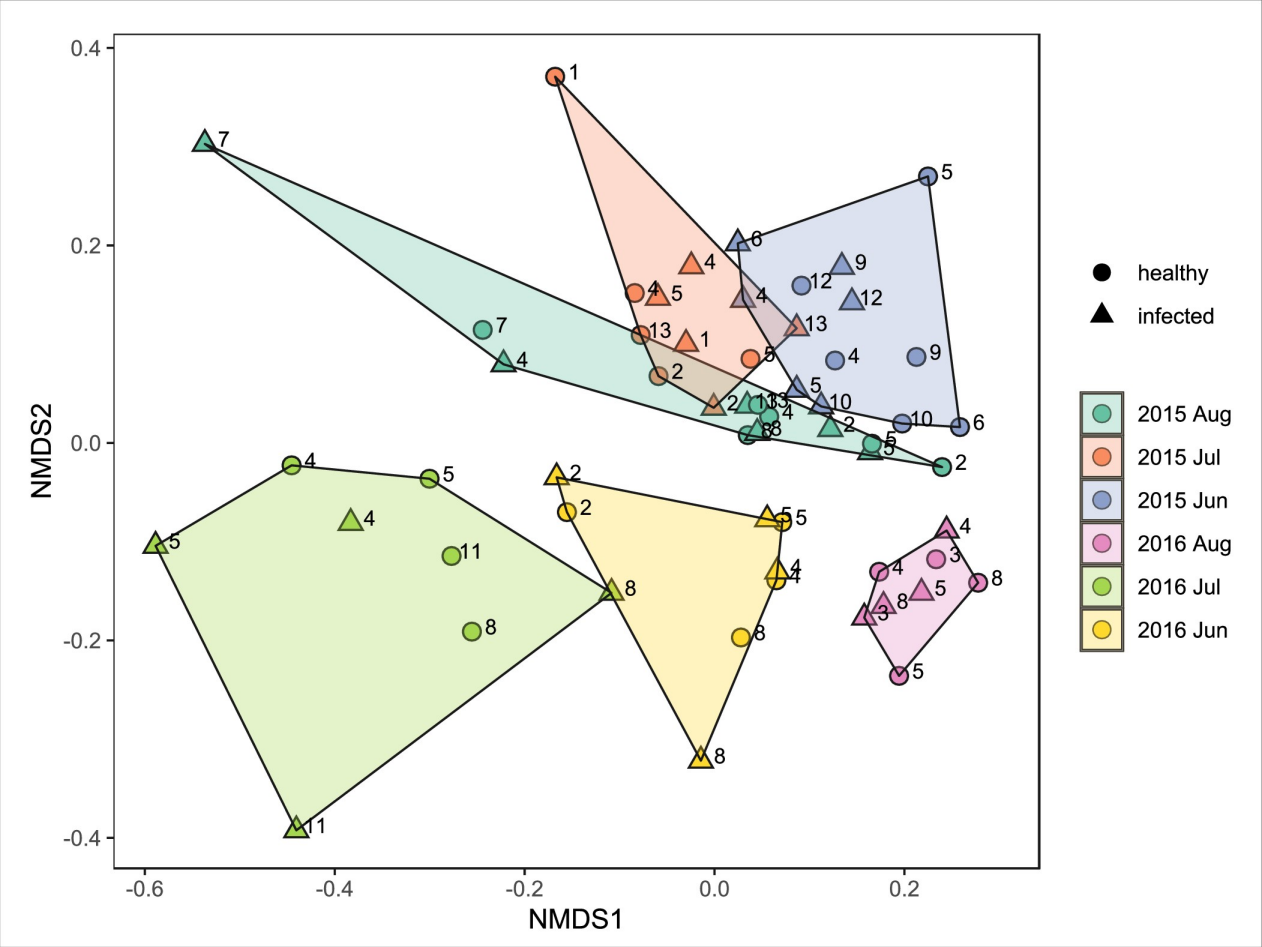
Biplot of Bray-Curtis dissimilarity of bacterial OTU abundances of teat skin swab samples from individual mammary glands by month of year and infection status of gland. Shape of points indicates infection status and color indicates sampling date. Paired infected and healthy samples collected from the same cow that visually cluster are indicated by an ellipse.

**Fig 5:**
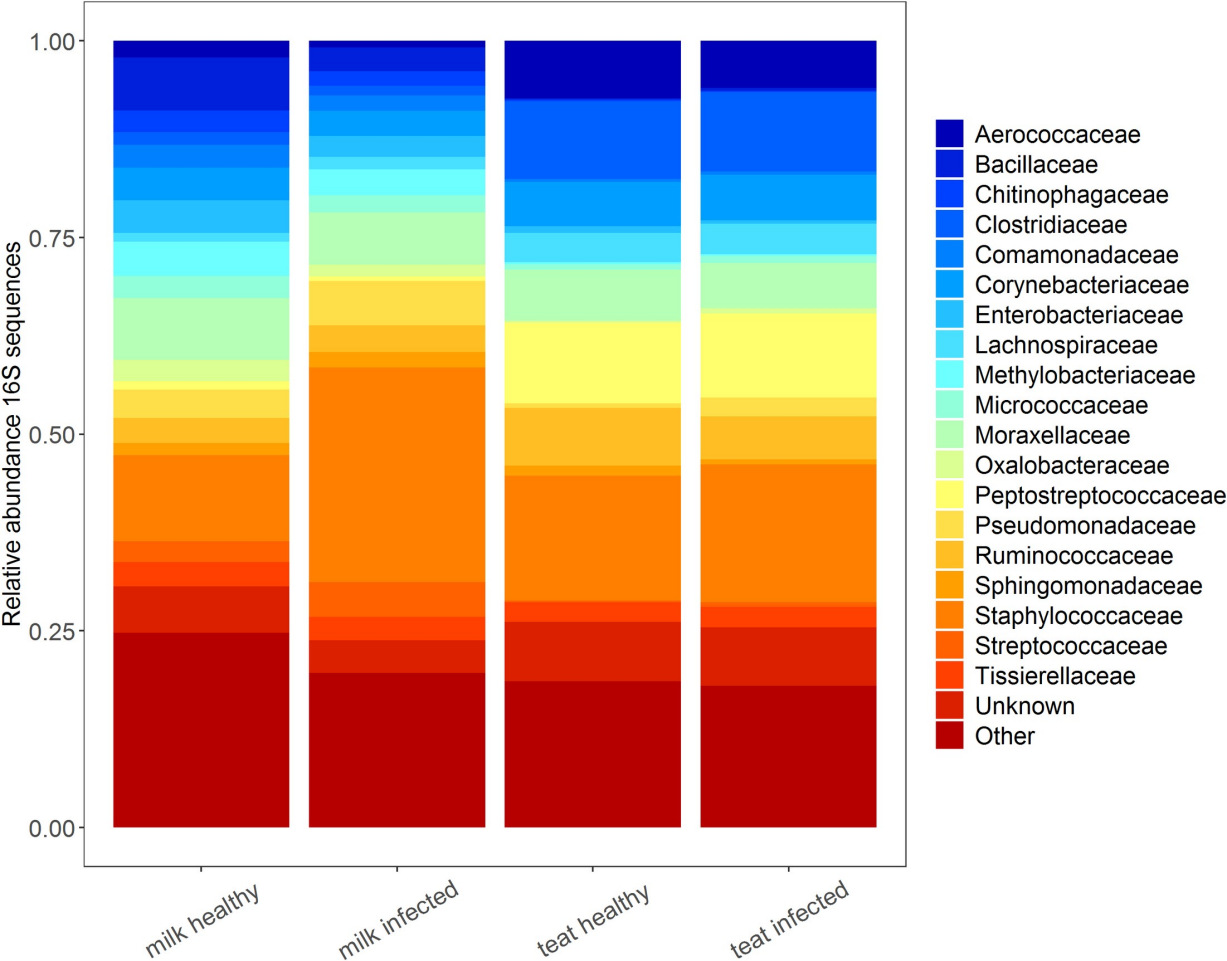
Twenty most relatively abundant categories of 16S rRNA gene sequence OTUs as arranged by bacterial family in each habitat state. “Unknown” category denotes unique OTUs that are unable to be classified at the family level, or potentially only as bacteria. “Other” category denotes those families that are not among the 20 most relatively abundant.

There was less variation in the composition of the bacterial microbiome among teat samples than among milk samples (*p* < 0.001) (Fig 2). Teat microbiomes were most similar if they were sampled on the same date (*p* = 0.005) (Fig 4), while milk microbiomes were not clearly affected by seasonal factors. However, 2015 and 2016 milk samples were distinct (*p* = 0.005). Some compositional differences among microbiomes were associated with an individual cow-level effect (teat *p* = 0.005, milk *p* = 0.015) (Fig 4). The milk microbiome was associated with infection state (*p* = 0.02), while healthy and infected teat end microbiomes were indistinct (*p* = 0.394) (Fig 5).

**Fig 6:**
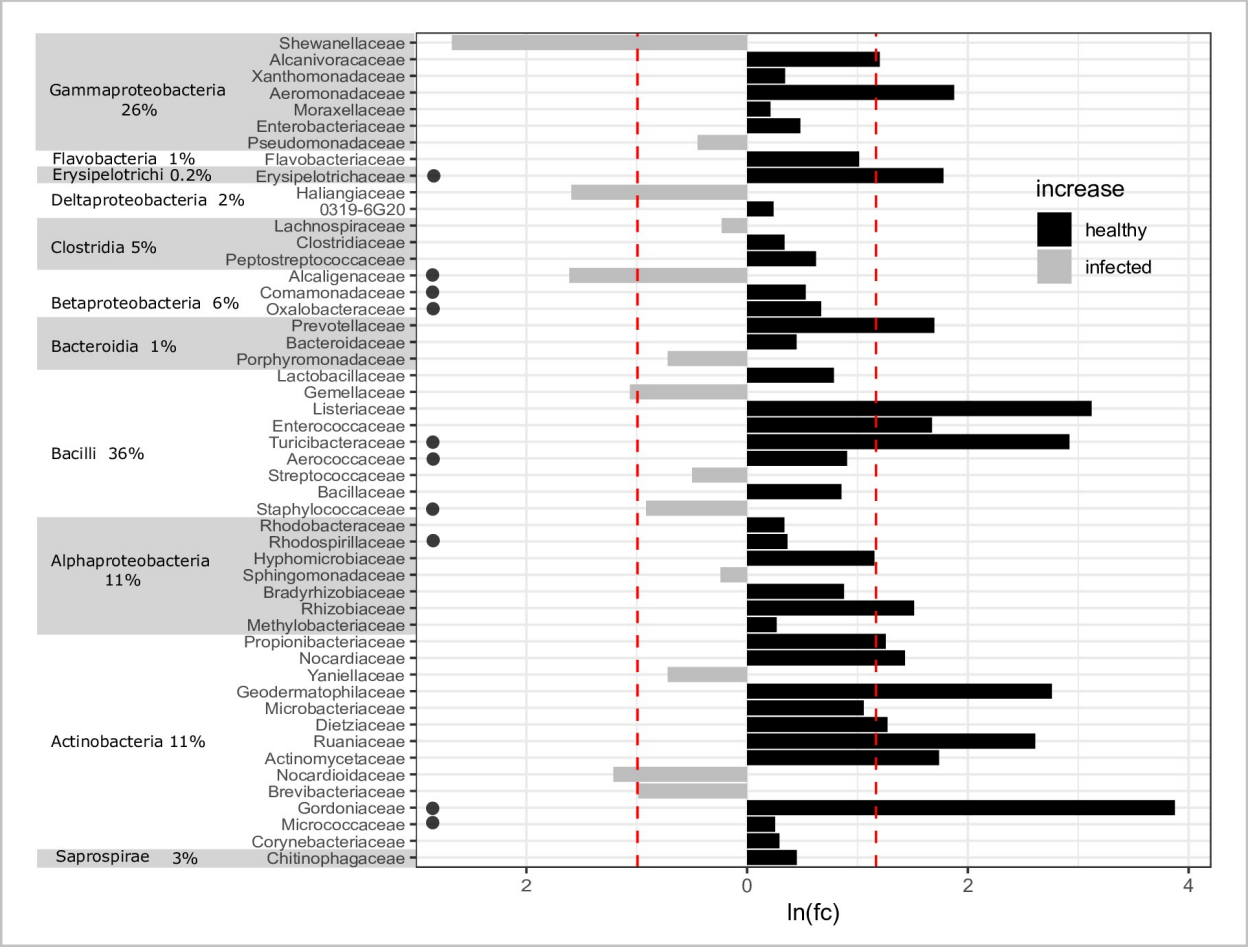
Fifty bacterial families that best differentiate microbiomes of healthy and infected milk. Difference was calculated by subtracting the lesser of the mean abundances from the greater. Families along the *y*-axis are sorted by class and labeled with the percent of overall milk microbiome sequences that each class represented. Color and direction of bar indicates whether healthy or infected glands was enriched in each taxa. *X*-axis represents natural log of fold change increase of abundance between infection status; a constant of 1.0 was added to avoid undefined numbers in the case that a family was absent. This occurred in the case of Shewanellaceae (absent in healthy), Gordoniaceae, and Listeriaceae (both absent in infected). Scale is positive in both directions from zero along axis. Red dotted line indicates mean log fold-change increase of presented families. Sequences that could not be classified to family are excluded. Dot to the right of taxon denotes *fdr* ≤ 0.9 via Kruskal Wallis test.

Families ≥ 2% relative abundance (RA) in both teat end and milk habitats in both years included Corynebacteriaceae, Moraxellaceae, Ruminococcaceae, and Staphylococcaceae (S4 Fig). Staphylococcaceae was both the most common family overall and best explained the observed differences in infection status of milk microbiomes. However, the greatest fold-change in abundance between milk infection states was observed for less abundant families that were only present in a single state (Fig 6). Two OTUs of *Staphylococcus* spp. dominated infected milk, an unidentified species (81%, OTU_001), and *S*. *sciuri* (19%, OTU_34895). *Pseudomonas* spp. and *Streptococcus* spp. were two orders of magnitude greater in infected compared to healthy milk. *Bacillus*, *Lactococcus*, and *Sediminibacterium* species were the reverse. Other genera exceeding 2% mean RA in both infection states of the milk microbiome included *Acinetobacter*, *Corynebacterium*, *Escherichia*, *Methylobacterium*, *Pseudomonas*, and *Staphylococcus*. Composition of teat microbiomes did not vary by infection status, characterized by an abundance of *Staphylococcus* and genus SMB53 (Clostridiaceae). Less common taxa included *Acinetobacter*, and unknown genera of the families Ruminococcaceae, Aerococcaceae, Peptostreptococcaceae, and Clostridiaceae.

### Habitat State: 16S rRNA sequences associated with infected and uninfected states for milk and teat skin

**Fig 7:**
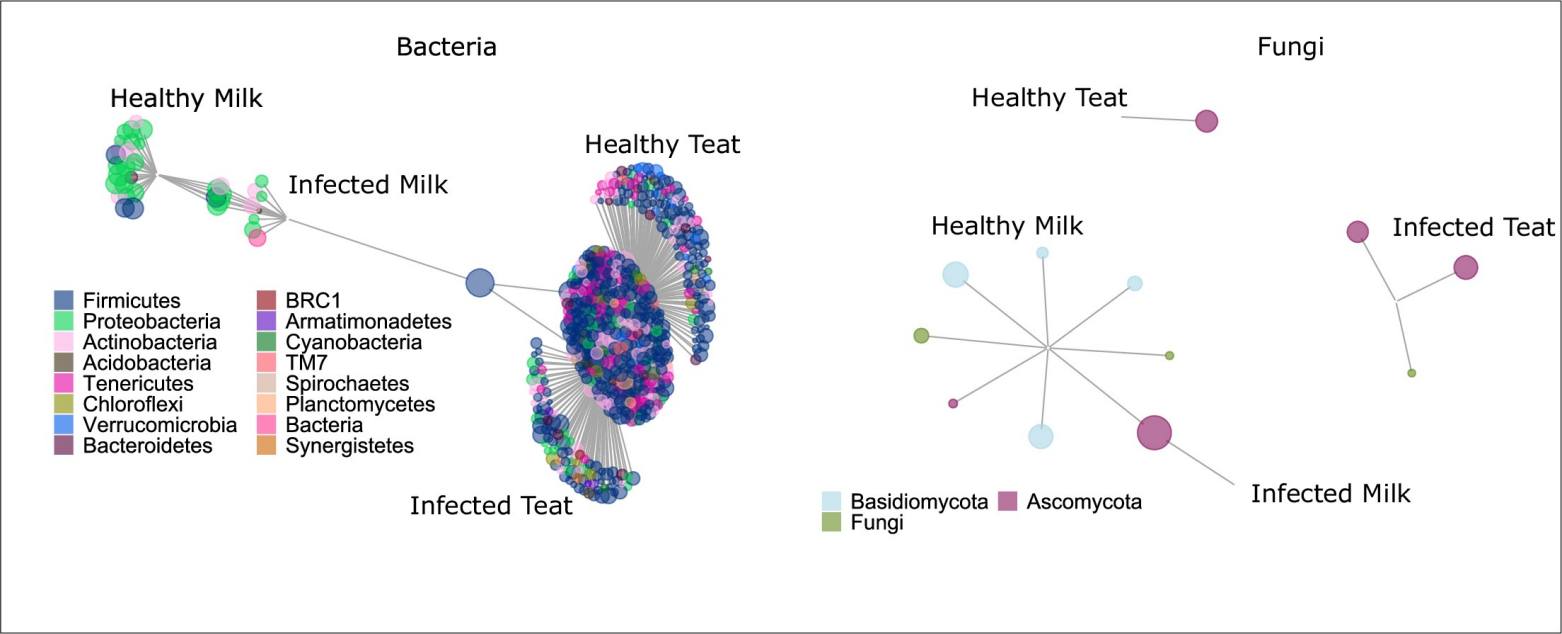
Bipartite networks of bacterial and fungal OTUs associated with one or multiple habitat states (infected milk, healthy milk, infected teat, healthy teat) in sampling year 2015. OTUs are included if chance of no association is *p* ≤ 0.05 as tested by indicator species analysis. Gray lines indicate association between habitat state(s) and OTUs (points). OTUs are colored by phylum and displayed on a log scale. The Firmicutes OTU connecting infected milk and teat is *Staphylococcus* OTU_001. The Ascomycota OTU connecting healthy and infected milk is *Penicillium olsonii*.

Most overlap in the composition of the milk and teat end microbiome occurred in infected glands. However, this trend was more prevalent in 2015 compared to 2016 (S5 Fig). *Staphylococcus* OTU_001 was the only OTU associated positively with both teat (healthy and infected) and infected milk habitat. In 2015, a diverse array of Proteobacteria was associated with the healthy milk microbiome (Fig 7). No OTUs were associated positively with both teat skin and healthy milk.

**Fig 8:**
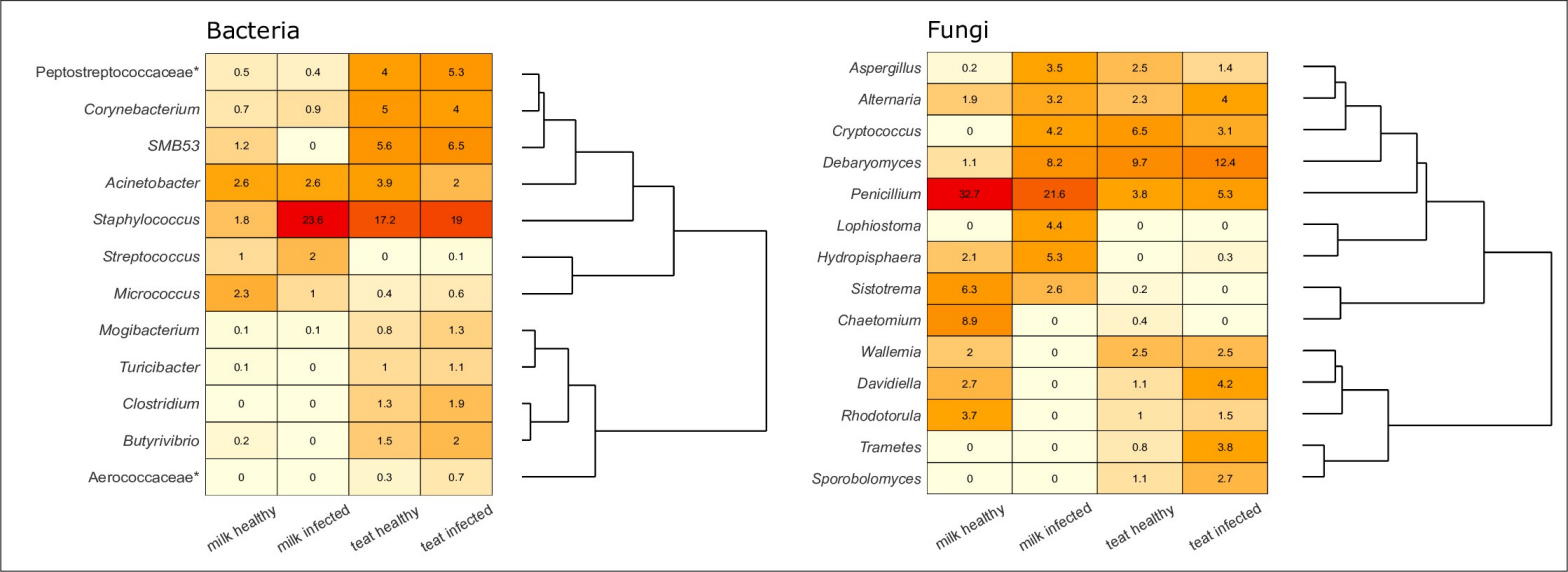
Heat maps of known fungal genera and core bacterial genera ≥ 1% relative abundance in year 2015. Illustrated bacteria were considered as members of the core. To be considered core, OTUs must first be observed in each habitat state in the global data set; of this group, OTUs are retained that were observed in all animals, in at least 50% of observations from that animal. This threshold retains OTUs that may only be seen in two of four habitat states, i.e., only milk or teat; or only infected milk and teat. No fungal genera met the core threshold. Genera in the accompanying dendrogram are grouped based on similarity of relative abundances in each category. Relative abundance within each group on *x*-axis is noted in each cell. Color is scaled from light yellow to red to visualize abundance (red is most abundant). Asterisk denotes OTU that was identified only to family level.

Relative abundances of the OTUs making up the core bacterial microbiome differed between infected and healthy milk (*p* = 0.001) but were similar between infected and healthy teat (*p* = 0.789) (Fig 8). Of shared genera, *Staphylococcus* was most abundant in each habitat state except healthy milk where *Micrococcus* and *Acinetobacter* were most abundant. Of *Acinetobacter*, the two OTUs shared between all habitat states exhibited opposite trends in milk. *A*. *lwoffii* was increased 2-fold in infected milk while *A*. *guillouiea* was increased 1.7-fold in healthy milk. Shared genera that were similarly abundant in healthy and infected teat samples, and trended toward an increased abundance in healthy milk, included *Micrococcus* and *SMB53* (*fdr* > 0.3). *Micrococcus* was present in milk from all cows and was more abundant in the healthy milk of 9 out of 12 cows. Genus *SMB53* was less common, present in 5 cows’ milk and greater in healthy milk from 3 cows.

At the family level, differences in composition of the microbiome between habitat were driven by both habitat and infection state. Families > 2% mean RA that differed in abundance (*fdr* ≤ 0.05) between milk and teat habitats in both infection states included Lachnospiraceae, Aerococcaceae, Clostridiaceae, and Peptostreptococcaceae (greatest in teat samples), and Enterobacteriaceae, Bacillaceae, and Methylobacteriaceae (greatest in milk samples). Families that differed between habitats of infected states included Comamonadaceae, Sphingobacteriaceae, Pseudomonadaceae, Veillonellaceae, and Lactobacillaceae (greatest in infected milk). Cytophagaceae were greatest in infected teat. Thirty-two families differed between the teat end and teat cistern milk only in healthy glands, of which 11 were absent in milk. Taxa with most significant difference included Alcaligenaceae (greatest in teat), Dehalobacteriaceae (absent in milk), and Brevibacteriaceae and Oxalobacteraceae (both greatest in milk).

### Fungal communities (ITS)

The fungal community was structured by year and habitat (Fig 2). Sequences were more abundant in teat than milk habitats and more variable in 2016 than in 2015 (Fig 1). Milk microbiomes contained < 50% of OTUs observed in teat microbiomes (*p* < 0.001), regardless of infection status, but more varied evenness of OTU abundances among individual samples (*p* = 0.004) (S6 Fig). In 2015, richness was greater in healthy glands (S6 Fig). Overall, 25 (3.2%) OTUs were common among all habitat states. Unique OTUs were greater than 5-fold more likely to be associated with teat than milk habitats.

Similar to bacteria, milk samples had more variation in the composition of the fungal microbiome than teat samples (*p* < 0.001; Fig 2) and were not sensitive to month of sampling, while teat microbiomes did vary by month (*p* = 0.001). In contrast to bacteria, the fungal microbiome was similar across infection states for both milk and teat habitats.

Families ≥ 1.5% RA in both teat end and milk habitats in both years included Trichocomaceae, Pleosporaceae, and an unknown family in order Saccharomycetales (S7 Fig). Dominant fungal genera in milk were often specific to gland. Genera including *Aspergillus*, *Basidiodendron*, *Bjerkandera*, *Cryptococcus*, *Lophiostoma*, *Pseudocercosporella*, *Scleroconidioma* and *Sordaria* were detected in approximately one infected gland with RA ranging 30% to 57%. Dominant genera only observed in approximately one healthy gland included *Chaetomium* and *Curvibasidium*. Of genera > 2% mean RA, *Debaryomyces*, *Penicillium*, and *Mortierella* were observed in 10 or more glands. *Debaryomyces*, consisting of a single species *D*. *prosopidis*, was greater in infected milk (*p* = 0.08). *Mortierella* and *Penicillium* were more often greater in healthy milk. *Mortierella* consisted of seven OTU’s, most commonly *M*. *exigua* and two other unknown taxa. *Penicillium* spp. were 46% greater in healthy milk and were most commonly *P*. *olsonii* (*p* = 0.1).

*Debaryomyces prosopidis* was also the most commonly occurring genus in the teat fungal microbiome; observed in 69% of samples. Other commonly occurring genera included *Cryptococcus*, *Caecomyces*, *Penicillium* and *Rhodotorula*.

### Fungal microbiome: Habitat comparison

**Fig 9:**
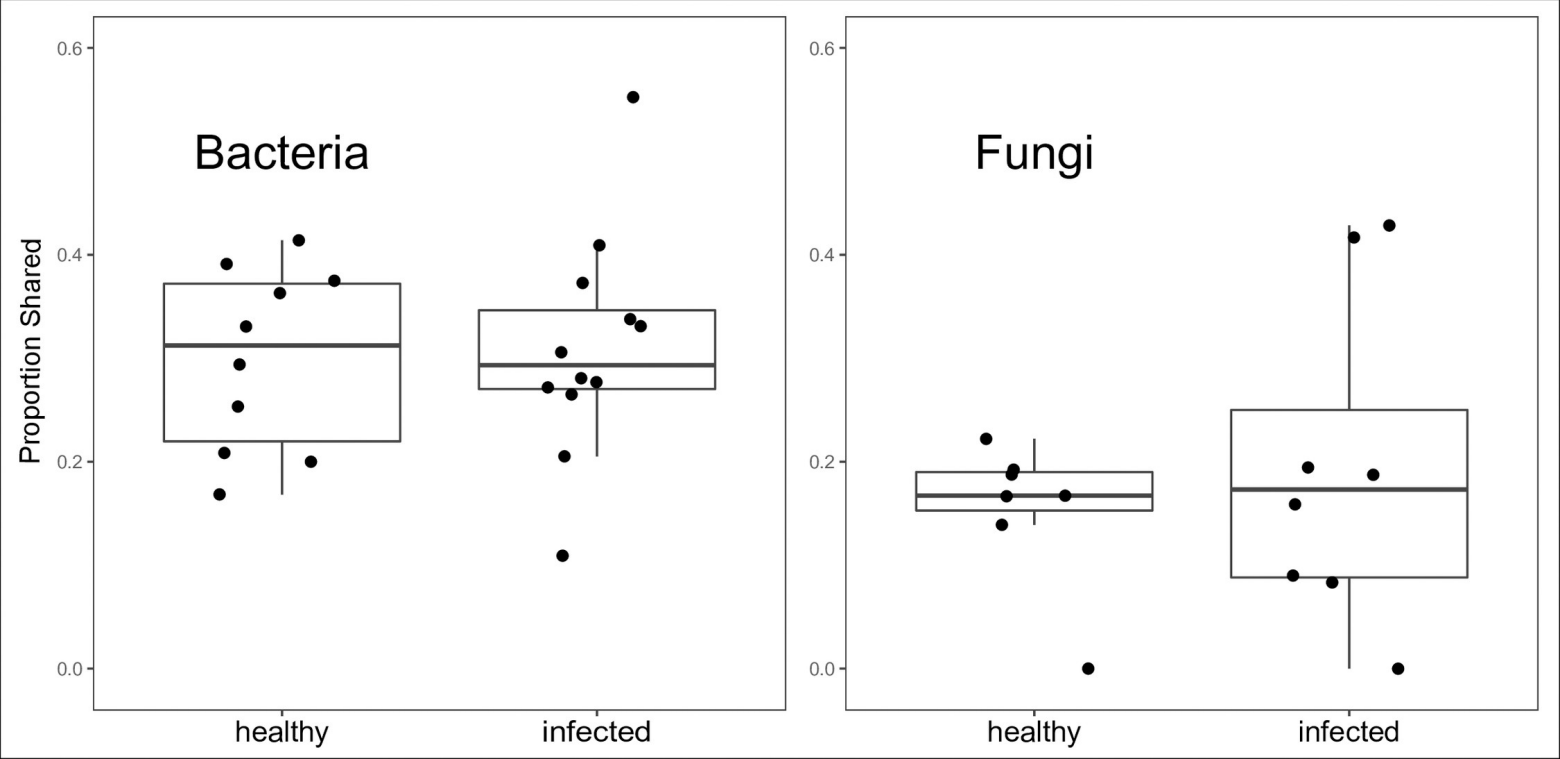
Proportion of milk OTUs that were also observed on the teat in 2015. Proportion of shared OTUs was calculated for each cow across all dates. Each point represents a unique cow. Least most outlier in fungal plot (cow 78) did not share any taxa between healthy and infected glands.

In both years combined, two taxa were shared between at least 30% of healthy milk, healthy teat, infected milk, and infected teat samples: *Debaryomyces prosopidis* and an unknown fungal taxon. In 2015, the proportion of taxa shared (observed at any abundance) between milk and teat varied widely by animal. The mean proportion of milk taxa that was also observed on the teat of each animal was similar between healthy and infected glands, but there was greater variation among infected glands than healthy glands (Fig 9). There were no core fungal OTUs. In 2015, *Debaryomyces prosopidis* and *Penicillium* spp. were often shared between infected teat and milk (Fig 8). Others included *P*. *chrysogenum* and *P*. *decumbens*. *Penicillium olsonii* was more abundant in milk than teat (*fdr* = 0.03), with the most observed in healthy milk. Indicator species analysis yielded fewer associations than bacteria, and networks were sparser; similar to bacteria, healthy milk had a more diverse array of associated OTUs than infected milk (Fig 7). The only OTU associated with both healthy and infected milk was *Penicillium prosopidis*. Healthy teat had no associated OTUs.

## Discussion

The results of this study support the dysbiosis hypothesis in the context of intramammary infections. A novel aspect of this work was the concurrent assessment of the teat skin microbiome, where there was no evidence that the teat end skin experienced dysbiosis associated with mastitis due to a subclinical intramammary infection. When compared to uninfected glands, a difference in the structure of the milk microbiome from infected mammary glands was characterized by dominance of a single *Staphylococcus* OTU and downward trend in OTU richness for both fungi and bacteria. The dominance of *Staphylococcus* spp. in milk from infected glands was consistent with the aerobic culture results and selection methods for infected glands, as the dominant intramammary pathogens on this farm were staphylococci. In contrast, and in agreement with other sequence-based assays of healthy glands, the healthy intramammary microbiome exhibited greater OTU richness and evenness than microbiomes of infected habitats [11]. Confirming the diversity analysis, indicator species analysis suggests more bacterial and fungal OTUs associated with healthy than infected milk samples.

The greater abundance of the genus *Staphylococcus* in infected intramammary milk samples does not prove that other taxa are reduced or impeded. It is possible that staphylococci were increased and the community remained stable; perhaps fewer OTUs were detected due to the greater likelihood of replicating and sequencing *Staphylococcus* spp. DNA. While decreased probability of replication likely explains some of the reduced diversity of the infected milk, this was not always the case. For example, some core milk genera, such as *Acinetobacter* and *Corynebacterium*, remained a similar proportion of the community in both infected and healthy milk despite the dominance of *Staphylococcus* DNA sequences. Likewise, a similar proportion of OTUs in both healthy and infected milk was detected exclusively in their respective habitat state. If a dominant sequence reduced the likelihood of rare DNA from being amplified, rare OTUs might be expected to be a lesser proportion of infected milk, yet this did not occur.

The dysbiosis concept is not without controversy, and we agree that equating the observation of microbial DNA in milk with the presence of an endogenous microbial community contradicts current understanding of the immunobiology of the mammary gland [6]. Another recent study, using an alternative extreme aseptic sampling technique and cisternal fine needle puncture, obtained quantities of microbial DNA from milk samples that yielded no bacterial colony growth in culture [3]. Others have used a teat cannula to bypass the teat canal and teat apex [22, 37]. These studies also reported microbial DNA in the teat cistern, though diversity was reduced compared to milk collected via traditional aseptic hand-stripping techniques. In this study, we adapted an alternative sampling technique of passing a catheter through a teat cannula to limit potential microbial contamination from the teat canal and the skin of the teat apex. Further research is required to understand the significance and relevance of the presence of microbial DNA and genes (i.e., the microbiome) in teat cistern milk and the potential importance of a teat apex and teat canal microbiota in mastitis epidemiology and control.

Results from sequencing studies are difficult to compare due to disparity of methods [3]. Consistent with our findings, the genera *Staphylococcus* and *Corynebacterium* are detected in many culture independent assays of the milk microbiota from both healthy and infected glands [3, 11, 13, 22, 37–39]. *Turicibacter* spp. and *Clostridium* spp. increased in composite samples of aseptically collected foremilk and teat apex interiors from healthy teats [12] and in aseptically collected milk [38], which mirrored the results from our study, though these genera were almost nil in milk collected by extreme aseptic methods [3] and more prevalent in teat samples, suggesting possible contamination of the milk from the teat skin in all studies. However, many discrepancies in differential abundance exist between our study and the previous, possibly due to the effect of the infecting organism [38], bedding type [3], days postpartum [39], and/or other factors that may influence the microbiota of the mammary gland.

There are few studies that use molecular techniques to describe the microbial ecology of the teat end skin. Like our study, these reports are characterized by a greater relative abundance of Firmicutes, Actinobacteria and Proteobacteria; specifically, *Staphylococcus* spp., *Micrococcus* spp., and *Acinetobacter* spp. were observed in teat samples from both infected and healthy glands [19, 40, 41]. We identified only one prior study using culture-independent methods to compare the teat microbiome of glands with different mastitis status [40]. Aligning with the results of our study, few differences were found among teat swab samples of healthy and infected glands, although richness was decreased in samples obtained from teat apexes of mammary glands with either subclinical or clinical intramammary infections compared to uninfected glands [40].

A novel aspect of our present study was the concurrent sampling of both teat and milk habitats in different infection states. There was relatively little overlap of OTUs between habitat states. Infected milk and infected teat samples were more similar to each other than were healthy milk and healthy teat samples. Most bacterial families were associated primarily with habitat, while some families varied between milk and teat in only the infected or the healthy state. The concept of core taxa enables a combined approach to OTU abundance and frequency of observation. Previous studies made inferences regarding the potential source of OTUs among ecologically linked sources of milk and teat microbiomes on dairy farms [42]. By concurrently observing both habitats, OTUs that are putatively transmitted from skin to milk or from milk to skin can be inferred and compared between infection states. *Staphylococcus* OTU_001 was common to all habitat states, although it was less dominant in healthy milk compared to infected, and not different for teat skin swabs from healthy and infected quarters. While this does not confirm directionality of transmission, it suggests that *Staphylococcus* OTU_001 either flows differently between skin and milk in the infected state compared to the healthy state or accumulates differently within each habitat state. For example, the flow of *Staphylococcus* OTU_001 between teat cistern and teat end may be similar in both states but the normal functioning of the healthy mammary immune response is able to lyse intramammary invaders more effectively, thus limiting intramammary bacterial growth rates [43] and reducing bacterial DNA in milk from the healthy gland. Both scenarios may explain the 13-fold increase of *Staphylococcus* spp. observed in the teat cistern milk samples of infected glands. While this is the most extreme case, all core taxa were found to be increased or reduced in at least one habitat state. For example, *Micrococcus* spp. and *Acinetobacter guillouiae* were commonly occurring taxa of similar abundance on skin of healthy and infected glands but elevated in milk from healthy glands. Variance in the putative sharing of taxa between skin and milk explained by infection status suggests that other refined molecular methods (e.g., shotgun metagenomics) might be used to explore the relationship between management practices and the teat skin microbiota.

A further novel aspect of this study is the culture independent characterization of the fungal microbiome of the udder. To the best of our knowledge, this is the first study to characterize fungal ITS amplicon sequence diversity from teat skin swab and cistern milk samples. Differential abundance of fungal DNA detected within the milk samples further exemplifies the recent call for an ecological interpretation of the intramammary space [20]. The yeast *Debaryomyces prosopidis*, though greatest in infected mammary glands, is not a noted mastitis pathogen. It is unknown what effect an elevated population has on a host animal, the greater microbial community, or mastitis pathogens. Likewise, the increased presence of *Penicillium* spp. in the healthy state raises questions about the effects of potential mechanisms of within- and across-domain interactions on and within the teat, including the production of antibiotics, on the resistance of the gland to infection or colonization.

The contrasting (though weak) trend in diversity of fungi between infection states, when compared to bacteria, appears to warrant further research. In 2015, fungal richness was less in milk from infected than healthy glands, and bacterial richness remained stable despite the emergence of a dominant OTU. In an ecological framework, a possible hypothesis is that infection disrupts fungal niche habitat, excluding teat taxa mostly observed in milk from healthy glands, and creates new opportunities for the establishment of genera such as *Aspergillus*, *Cryptococcus*, and *Debaryomyces*, which were observed mostly in milk of infected glands. This hypothesis is also supported by greater similarity of the proportion of milk OTUs observed on teats from healthy glands through time when compared to the proportion shared between habitats of infected glands (Fig 9). The tight clustering around the mean richness of healthy glands suggests a more stable interaction between teat and milk communities in the healthy state, while the infected milk is less stable, variably sharing a much increased or decreased proportion of OTUs with the teat. Notably, this trend did not emerge in bacterial data, again raising questions about domain level mechanisms of microbe-host interactions.

While it was clear that some variation in the microbiome was associated with factors related to the sampling date, one might have expected to see similar trends emerge through the same months in both years. This was not the case, as much of the variation in the global microbiome was observed between years. Some of the variation between years may be attributable to the commencement of teat-dipping between year 1 and year 2, which was an unexpected management change implemented on this commercial farm. Unfortunately, it is impossible to reach conclusions because year and teat-dipping are exactly confounded. That said, fewer sequences and OTUs detected in 2016 (during teat-dipping) align with the objective of teat-disinfection and seem to confirm the intended effect, possibly meriting further research, especially in the light of the increase of fungal sequences and opposing decrease of bacterial sequences on 2016 teat end skin. These findings contrast with a study of pasture-fed cows in which number and diversity of bacterial species correlated positively with low somatic cell counts when receiving no teat preparation compared to time periods when the same cows received pre-milking teat preparation and disinfection [42]. During the months of our study in both years, the cattle grazed on perennial grass-legume pastures on a 24-h rotational grazing regime similar to the study of Doyle et al., [42]. However, Doyle et al. concluded that the housing environment (i.e., “herd habitat”) was a significant driver of milk and teat skin microbial community composition and that teat prep had a more limited impact on raw milk and mammary microbiota. In the two years of our study, housing environment was essentially the same, although we did not characterize potential differences in weather (temperature, rainfall, humidity, etc.) in the summers of 2015 and 2016 on this farm. Therefore, while we found a difference in milk and skin microbiota across months and years, these data do not provide insight on the effect of teat disinfection or weather variation on the milk microbiome in this pasture-based dairy herd.

## Conclusion

Exploring farm management practices to foster a commensal mammary microbiome that limits infection risk or improves the return to a stable, healthy habitat state is an important applied research goal. While the link between a teat skin microbial community structure and risk of intramammary infection remains unknown, our study suggests that infection state effects transference or accumulation of microbial DNA between cistern milk and teat skin. Increased sharing of an OTU between habitats in the healthy state may be indicative of a beneficial organism or functional group. Oppositely, increased sharing in the healthy state may be a predictor of future infection or a residual imprint of prior infections or immune activity. The strong effect of sampling month suggests microbial communities change through time in response to seasonality or other factors. Future comparisons should seek controlled designs that accommodate temporal dynamics of ecosystems and a gradient of infection states.

## Supporting information

S1 Fig Bacterial taxa observed in control samples. Colors are scaled by read counts (blue is least abundant, red is most abundant).
(TIF)

S2 Fig Fungal taxa observed in control samples. Colors are scaled by read counts (blue is least abundant, red is most abundant).
(TIF)

S3 Fig Rarefaction curves for 16S and ITS rRNA genes in years 2015 and 2016. Each sample is randomly subset stepwise without replacement to represent the relationship between sequencing depth and OTU richness.
(TIF)

S4 Fig 100 bacterial taxa with greatest median relative abundance. Colors are scaled within habitat state (blue is least abundant, red is most abundant. Taxa are sorted by higher classification.
(TIF)

S5 Fig Coinertia analysis. Quadrants compare global similarity (RV) of habitats in both healthy and infected state in 2015 and 2016. RV is bound between 0–1, closer to 1 indicates greater similarity. Lower two plots in each quadrant represent ordination of OTUs along first two axes that explain most variation in both data sets. Upper plot in each quadrant represents paired samples (teat and milk from same animal on the same date). The points represent teat samples and the arrowhead represent milk samples. Paired samples are connected by a line. Shorter linear distance between paired samples indicates greater similarity.
(TIF)

S6 Fig Fungal OTU diversity as represented by richness and evenness of OTUs in each habitat state and year. Richness was calculated as number of OTUs; evenness was measured using Pielou's J (J = Shannon/log(richness)). Index values closer to 1.0 indicate increasingly even distributions of OTU abundances.
(TIF)

S7 Fig 100 fungal taxa with greatest median relative abundance. Colors are scaled within habitat state (blue is least abundant, red is most abundant. Taxa are sorted by higher classification.
(TIF)
