# Supplementary figures and images for: Mammary microbiome of lactating organic dairy cows varies by time, tissue site, and infection status
Source: PLoS One. 2019 Nov 14;14(11):e0225001. doi: 10.1371/journal.pone.0225001 (PMC6855453; doi:10.1371/journal.pone.0225001)

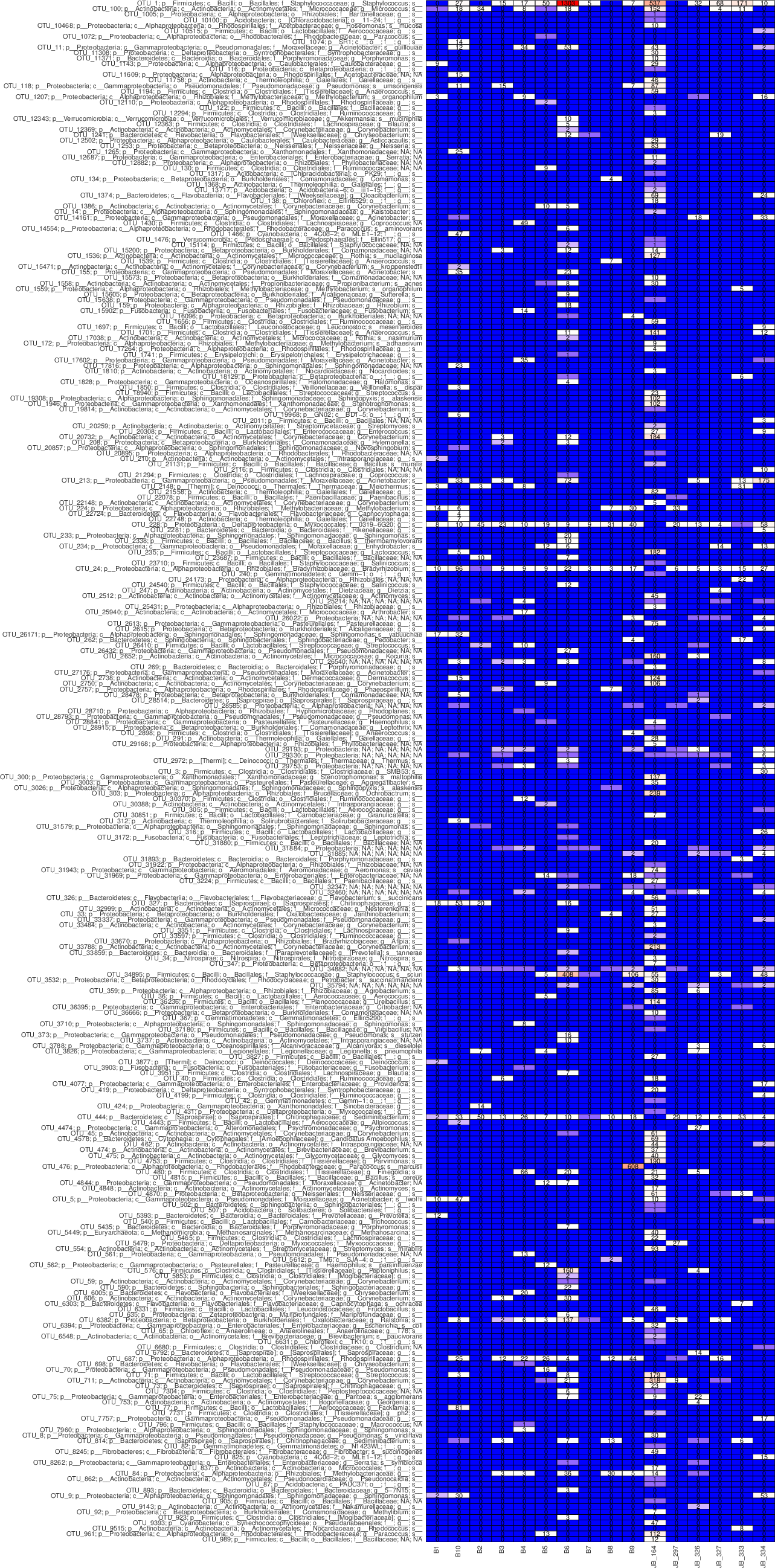

Supplement: S1 Fig — Colors are scaled by read counts (blue is least abundant, red is most abundant). (TIF) [file pone.0225001.s001.tif]

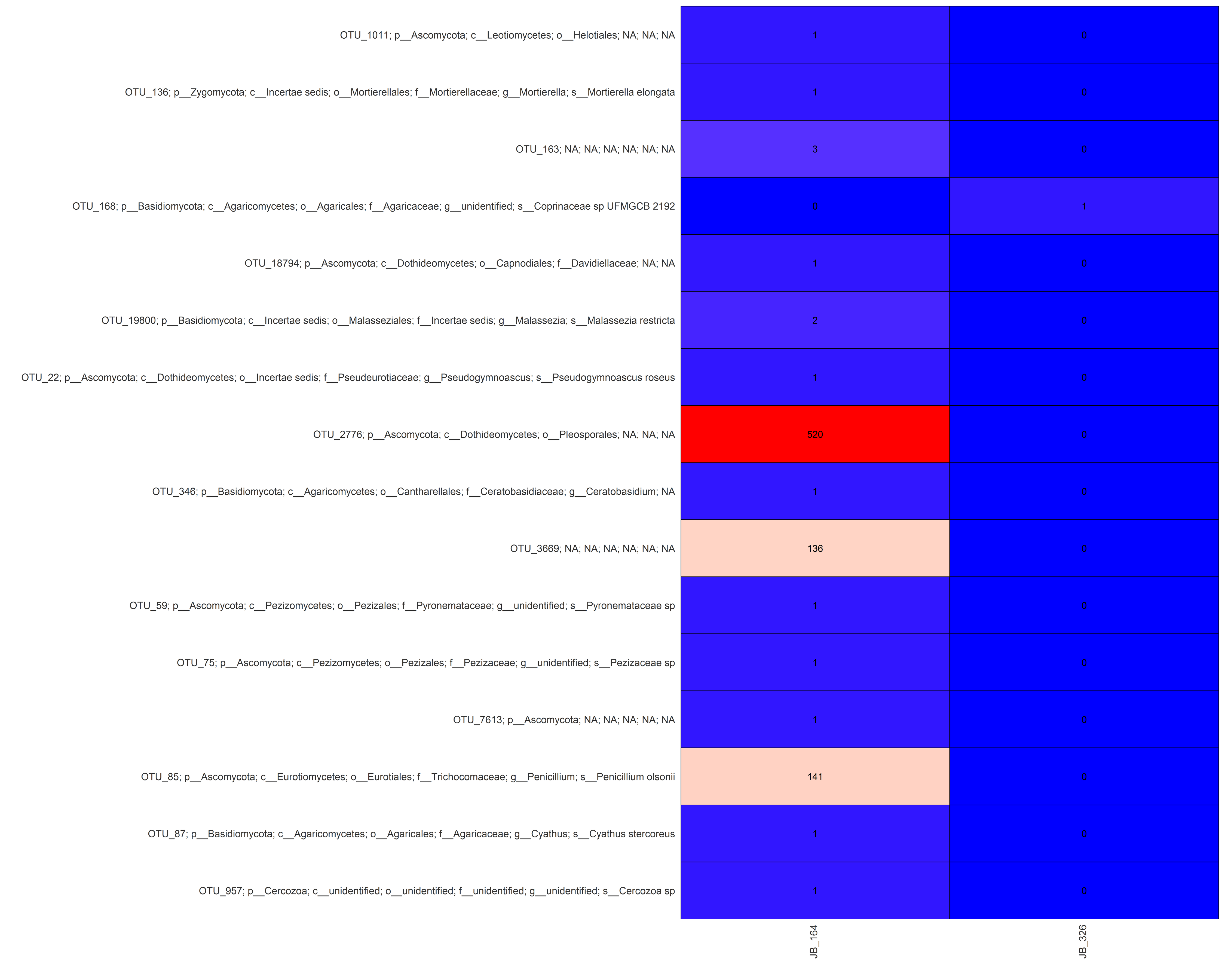

Supplement: S2 Fig — Colors are scaled by read counts (blue is least abundant, red is most abundant). (TIF) [file pone.0225001.s002.tif]

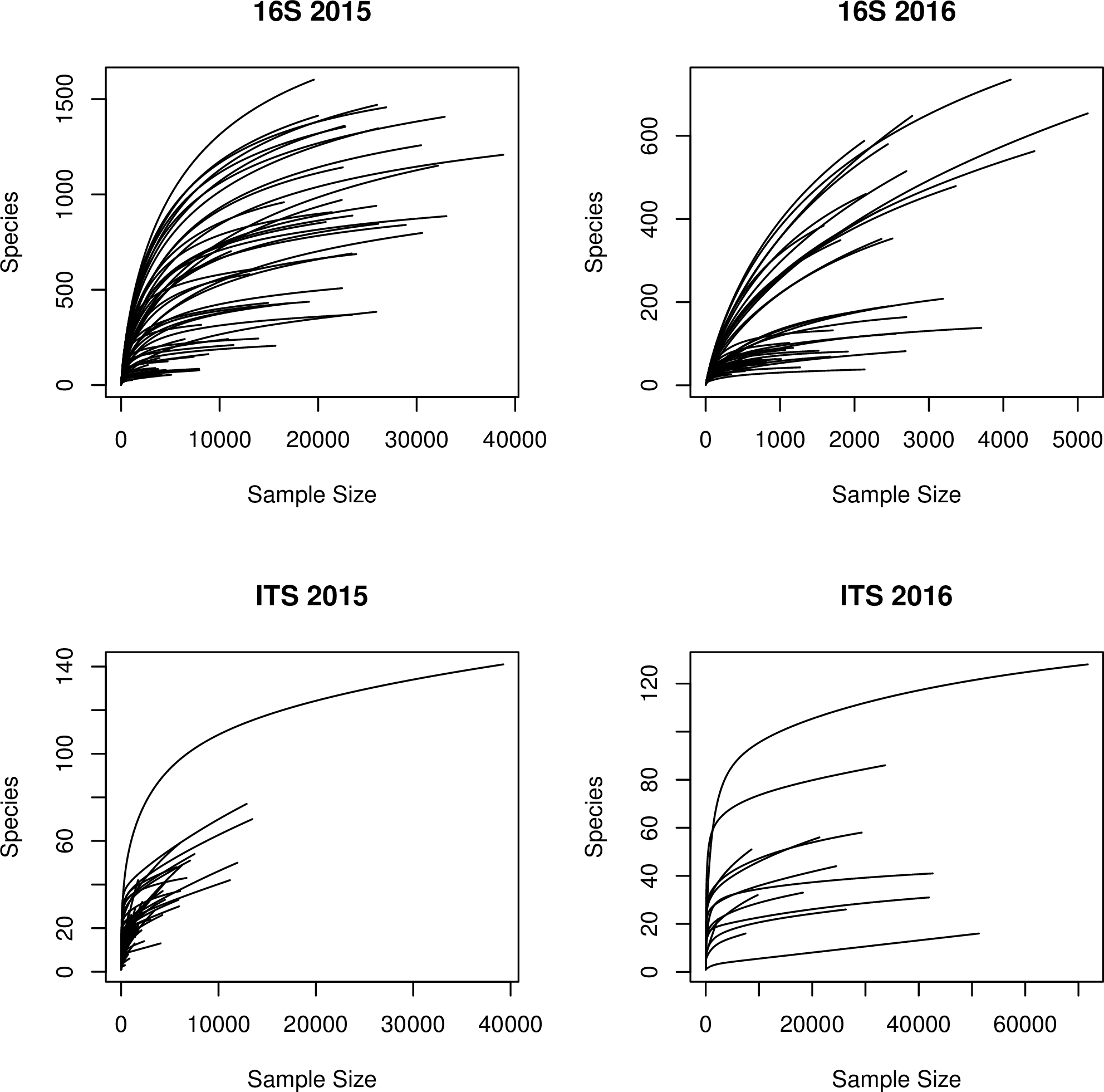

Supplement: S3 Fig — Each sample is randomly subset stepwise without replacement to represent the relationship between sequencing depth and OTU richness. (TIF) [file pone.0225001.s003.tif]

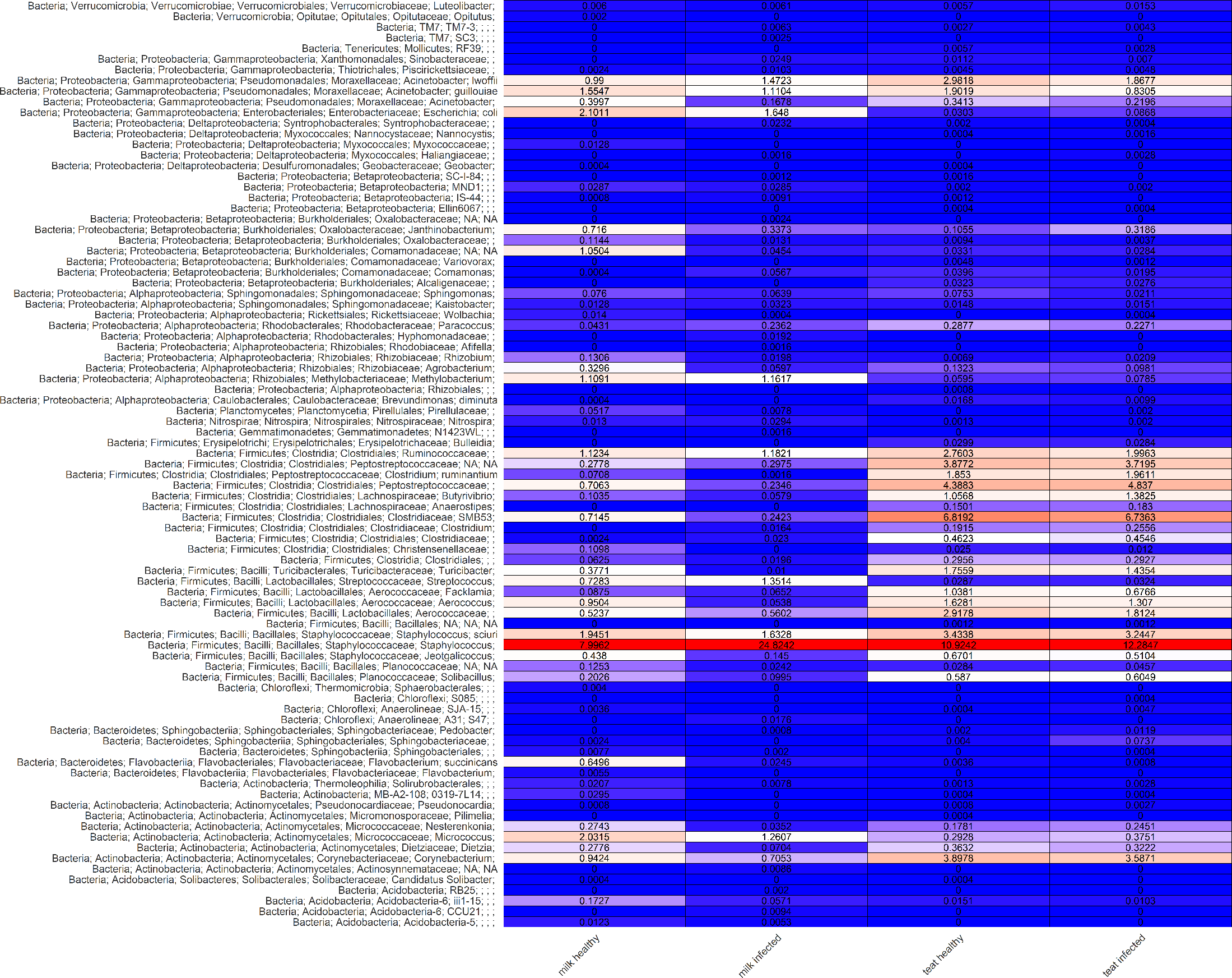

Supplement: S4 Fig — Colors are scaled within habitat state (blue is least abundant, red is most abundant. Taxa are sorted by higher classification. (TIF) [file pone.0225001.s004.tif]

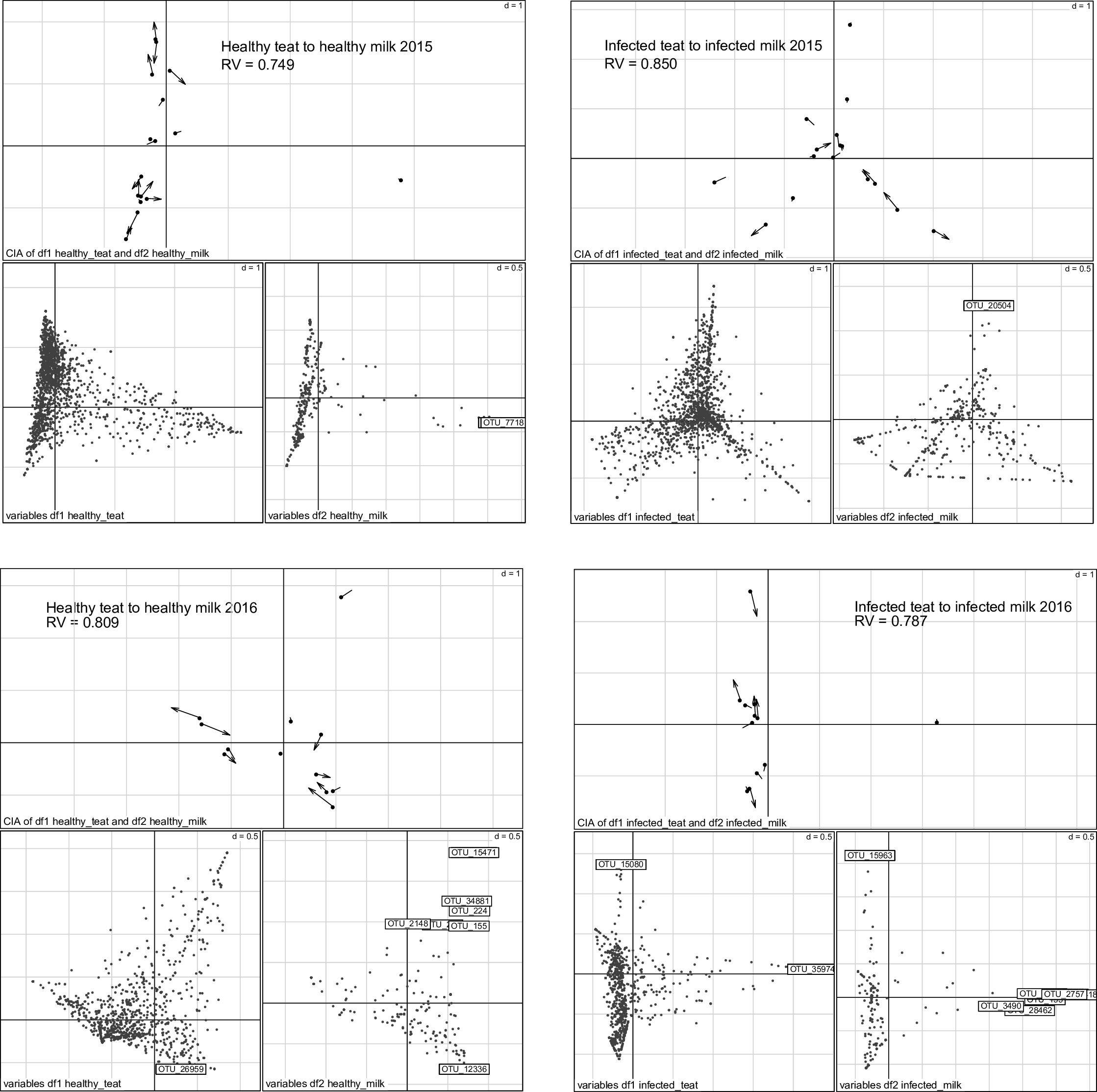

Supplement: S5 Fig — Quadrants compare global similarity (RV) of habitats in both healthy and infected state in 2015 and 2016. RV is bound between 0–1, closer to 1 indicates greater similarity. Lower two plots in each quadrant represent ordination of OTUs along first two axes that explain most variation in both data sets. Upper plot in each quadrant represents paired samples (teat and milk from same animal on the same date). The points represent teat samples and the arrowhead represent milk samples. Paired samples are connected by a line. Shorter linear distance between paired samples indicates greater similarity. (TIF) [file pone.0225001.s005.tif]

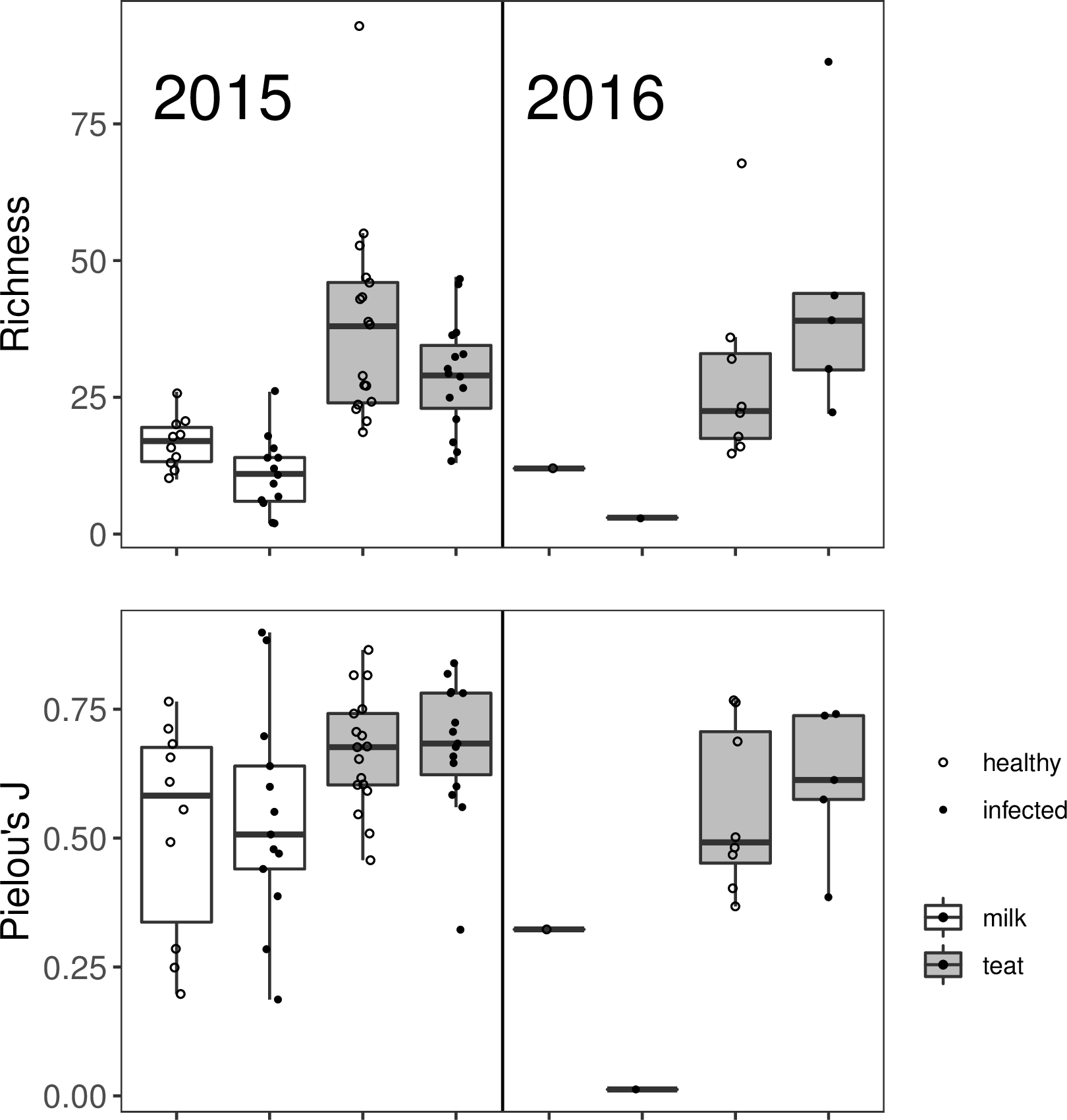

Supplement: S6 Fig — Richness was calculated as number of OTUs; evenness was measured using Pielou's J (J = Shannon/log(richness)). Index values closer to 1.0 indicate increasingly even distributions of OTU abundances. (TIF) [file pone.0225001.s006.tif]

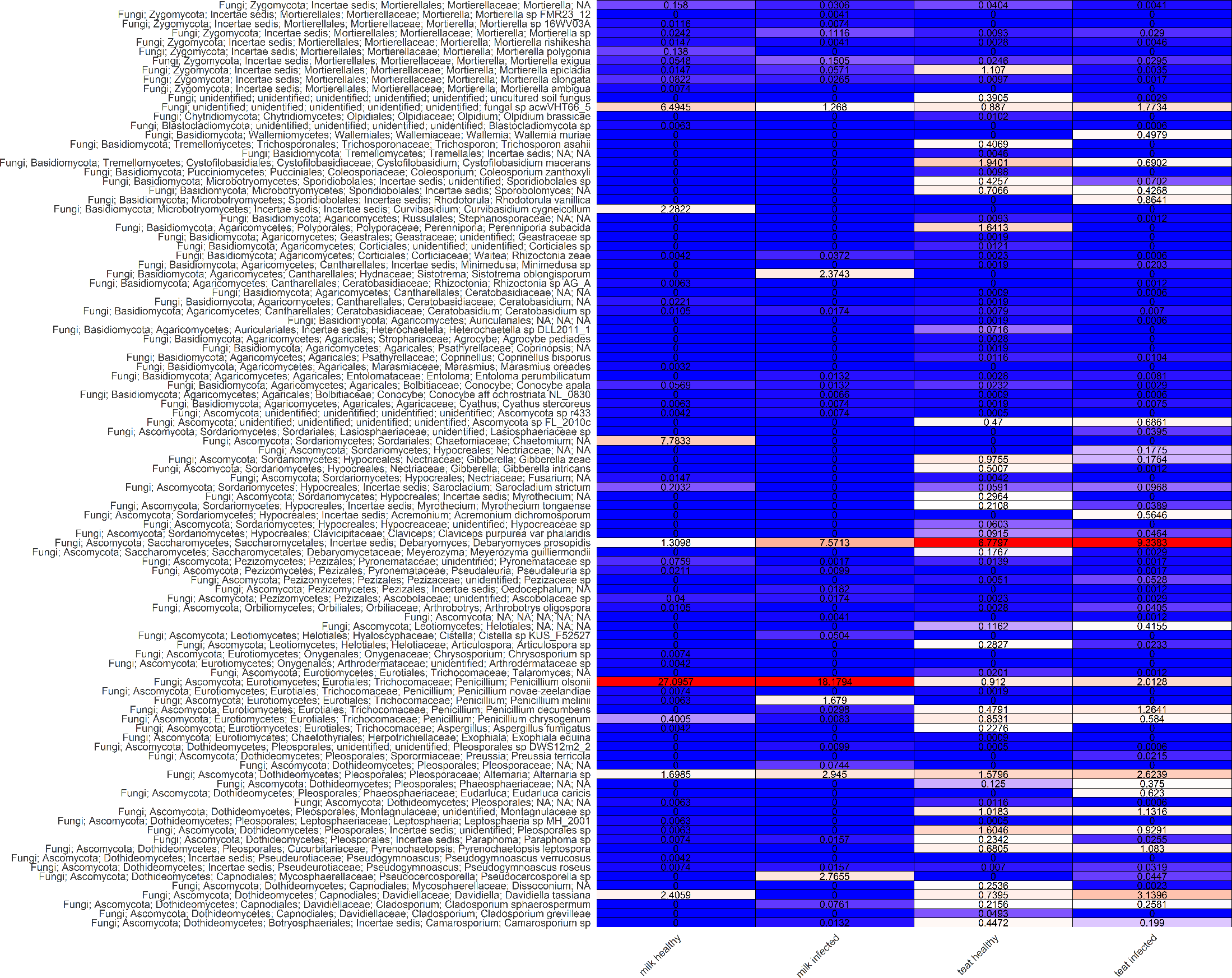

Supplement: S7 Fig — Colors are scaled within habitat state (blue is least abundant, red is most abundant. Taxa are sorted by higher classification. (TIF) [file pone.0225001.s007.tif]
